# Control of Rest:Activity by a Dopaminergic Ultradian Oscillator and the Circadian Clock

**DOI:** 10.3389/fneur.2017.00614

**Published:** 2017-11-27

**Authors:** Clément Bourguignon, Kai-Florian Storch

**Affiliations:** ^1^Integrated Program in Neuroscience, McGill University, Montreal, QC, Canada; ^2^Douglas Mental Health University Institute, Montreal, QC, Canada; ^3^Department of Psychiatry, McGill University, Montreal, QC, Canada

**Keywords:** dopaminergic ultradian oscillator, biological rhythms, circadian clock, dopamine transporter, rest:activity

## Abstract

There is long-standing evidence for rhythms in locomotor activity, as well as various other aspects of physiology, with periods substantially shorter than 24 h in organisms ranging from fruit flies to humans. These ultradian oscillations, whose periods frequently fall between 2 and 6 h, are normally well integrated with circadian rhythms; however, they often lack the period stability and expression robustness of the latter. An adaptive advantage of ultradian rhythms has been clearly demonstrated for the common vole, suggesting that they may have evolved to confer social synchrony. The cellular substrate and mechanism of ultradian rhythm generation have remained elusive so far, however recent findings—the subject of this review—now indicate that ultradian locomotor rhythms rely on an oscillator based on dopamine, dubbed the dopaminergic ultradian oscillator (DUO). These findings also reveal that the DUO period can be lengthened from <4 to >48 h by methamphetamine treatment, suggesting that the previously described methamphetamine-sensitive (circadian) oscillator represents a long-period manifestation of the DUO.

## Introduction

Many species on earth have evolved a self-sustaining timing system, likely to facilitate robust 24-h rhythms in physiology and behavior despite non-24-h variations in the environment. This timing system, the circadian clock, has been studied in detail over the past decades, uncovering its cellular and molecular basis (1, 2). In addition to 24-h variations, there are also numerous accounts of cyclic changes in physiology and behavior with periods much shorter than 24 h, i.e., in the ultradian range. Ultradian rhythms with periods of 2–6 h have been reported in the context of locomotion, sleep, feeding, body temperature, and serum hormones levels, in species from the fruit fly to humans (3–13). However, in sharp contrast to circadian rhythms, the biological substrate and mechanistic basis of ultradian rhythm generation has remained elusive.

### Ultradian Behavior in Voles and Mice: Hourglass vs. Oscillator

**Figure 1:**
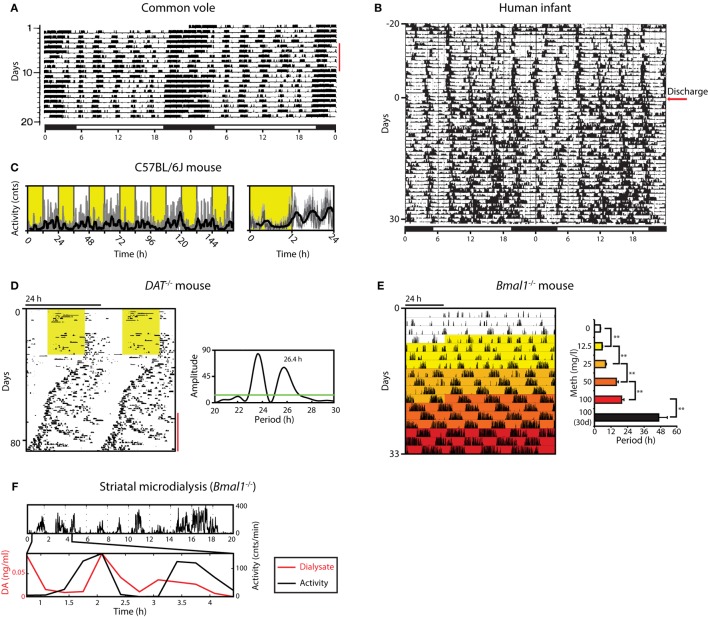
Ultradian rhythms and their manipulation from voles to humans. **(A)** Locomotor activity (LA) rhythms in the common vole in the presence of a running wheel; red bar indicates days when the wheel was blocked; bar on top indicates periods of lights on (white) and off (black); adapted from Ref. (16) with permission. **(B)** Activity record of a preterm infant based on ankle-actigraphy; arrow indicates day of hospital discharge; adapted from Ref. (8) with permission. **(C)** Recording of ambulatory activity in the mouse by telemetry implants; right, average daily activity based on primary data shown on the left; yellow shading indicates lights on. **(D)** Running wheel activity of a DAT^−/−^ mouse; yellow area, lights on; red bar indicates the emergence of a second rhythmic component, supported by periodogram analysis (right). **(E)** Gradual ultradian locomotor period lengthening by increasing methamphetamine concentration in the drinking water of Bmal1^−/−^ mice in constant darkness. **(F)** Extracellular dopamine measured by microdialysis in the striatum fluctuates synchronously with ultradian LA in Bmal1^−/−^ under constant dim red light. Graphs shown in D, E, F are adapted from Ref. (20).

While ultradian range rhythms are often found to be labile when compared to circadian/diurnal rhythms (14, 15), a particular overt and robust expression of ultradian behavior is exhibited by the common vole (*Microtus arvalis*) (Figure 1A) (5). This is thought to be due to evolutionary pressures resulting in the emergence of synchronous ultradian day time foraging as a strategy to reduce predation risk: by emerging from the burrows during the daytime every 2–3 h in synchrony, the voles are less likely to fall prey to a kestrel (5). Ultradian rhythm expression does not require the circadian timer as rhythms persist in the vole after lesioning of the suprachiasmatic nucleus (SCN), the central circadian pacemaker site (16). While such ultradian behavior could be the output of a discrete rhythm generator, it may as well be driven by physiological demand, such as energy depletion or sleep debt. However, food, water, or sleep deprivation does not affect ultradian locomotor activity (LA) of the vole in substantial ways (5, 17). For instance, if—in the laboratory cage setting—food access is blocked, the voles still engage the food access bar at the same ultradian period as under conditions of *ad libitum* food access (17). Equally, forced lengthening of the active phase by rest deprivation does not lead to a proportional increase in subsequent rest time, which consequentially would result in ultradian period lengthening (17). It appears instead that sleep rebound is facilitated by an increased rest:activity ratio within a given ultradian cycle, instead of changing the cycle length *per se*. Taken together, these data argue against a role of behavioral output to define or regulate ultradian period but favor an endogenously generated, self-sustained oscillatory process that does not require a “driver,” as would be the case if the ultradian rhythmicity is based on an hourglass mechanism (18, 19).

In contrast to voles, ultradian components in LA are less overt but still detectable in circadian intact laboratory mice, exhibiting periods of 3–5 h (7, 20) (Figure 1C). Elimination of the master circadian pacemaker by SCN lesion or genetic manipulation renders them readily observable, however, murine ultradian locomotor rhythms are typically less robust compared to the vole, exhibiting a wider frequency range with substantial inter- but also intra-animal variation (20–22).

### Ultradian Activity in Humans

Overt ultradian behavior has been also reported for human infants (8, 23–25). Activity recordings based on ankle-actigraphy revealed clear ultradian rhythmicity in preterm infants regardless of whether they were exposed to constant dim light or a 24 h light:dark (LD) cycle (Figure 1B) (23). While the periodic activity bouts could potentially result from rhythmic interference by nursing staff, sleep diary recordings of term infants by mothers who breastfed at the infant’s will also revealed ultradian patterns in feeding and sleep (25). Of note, these ultradian patterns within the first few months of postnatal life were observed in the majority of the infants tested. These reports also suggest that—in humans—the circadian and/or diurnal control of sleep:wake rhythmicity only establishes over the course of weeks to months postnatally, thereby permitting an “unobstructed” view on ultradian rhythms in the 2–6 h range during this early postnatal period. The actigraphy and sleep diary data suggest that once the circadian and/or diurnal control of sleep:wake is established, both the ultradian and 24-h rhythmic components integrate in a harmonic fashion (see, e.g., Figure 1B, bottom half of the record) (8). The resulting compound pattern that is distinctly observable in some cases supports the idea that an ultradian rhythm generator has perhaps evolved or has been evolutionary adopted to promote social synchrony in gregarious species, precipitating for instance a frequency of three major meals per day, which seems to dominate the temporal structure of human food intake.

## A Case for a Dopaminergic Oscillator Driving Ultradian Behavior

### Monoamines and the Ascending Arousal Pathway

The monoamines histamine, norepinephrine, serotonine, and dopamine have all been associated with the ascending arousal pathway and are considered to be key elements of wakefulness promotion (26, 27). Interestingly however, genetic manipulation of monoamine levels by disrupting their biosynthesis or reuptake systems has only relatively mild effects on LA (28–32) except in the case of dopamine (33, 34). DA reuptake blockade (35) leads to a profound hyperlocomotor (33) phenotype and abolishing dopamine synthesis by tyrosine hydroxylase gene disruption selectively in DA neurons leads to an almost complete loss of spontaneous LA (34, 36). Thus, among the monoamines associated with the ascending arousal pathway, dopamine has the strongest link to LA, which is highly associated with the wake state (37).

### DAT Removal Lengthens Ultradian Period

When running wheel activity is monitored long-term, mice deficient of the dopamine transporter *(DAT;* official gene name, *Slc6a3)* exhibit less consolidated, rather erratic activity that nevertheless remained largely confined to the dark period of the LD cycle when compared to wild-type littermates (Figure 1D) (20). However, upon switching to constant darkness (DD), periodogram analysis revealed the emergence of a second component of rhythmic activity that persisted over several cycles with a period longer than 24 h, while the primary or circadian component exhibited periods below 24 h as expected for endogenous circadian pacemaking of the C57BL/6J laboratory mouse strain that served as genetic background for the *DAT^−/−^* mouse line (Figure 1D). Further examination revealed that this second component does not result from a phase dissociation within the SCN clock cell ensemble, which has been shown to account for the split locomotor rhythm observed in hamsters exposed to constant light (38), or for the two component pattern in rats exposed to a 22 h LD cycle (39). If the second, >24 h component observed in *DAT^−/−^* animals indeed results from the very oscillator that normally accounts for ultradian activity, then upon elimination of the circadian pacemaker, these mice would be expected to show lengthened ultradian activity cycles. Indeed, when running wheel activity of *DAT^−/−^* mice is monitored in constant darkness following SCN-lesion or genetic disruption of the circadian clock, a profound lengthening of the ultradian locomotor period is observed, from the typical 2- to 4-h period to ~12 h (20).

### Striatal Dopamine Fluctuates in Step with Ultradian Activity

It was further found that extracellular dopamine levels in the striatum of *Bmal1^−/−^* mice kept in DD fluctuate in synchrony with ultradian LA (Figure 1F), and that extracellular levels of striatal DA strongly correlate with ultradian period (20). Together, these findings are in support of dopamine acting as an ultradian oscillator output and at the same time as a period determinant, arguing for a central role of dopamine in the ultradian rhythm generation process. Hence, the name dopaminergic ultradian oscillator (DUO) was coined (20).

## DA Neurons, Site of Ultradian Rhythm Generation?

**Figure 2:**
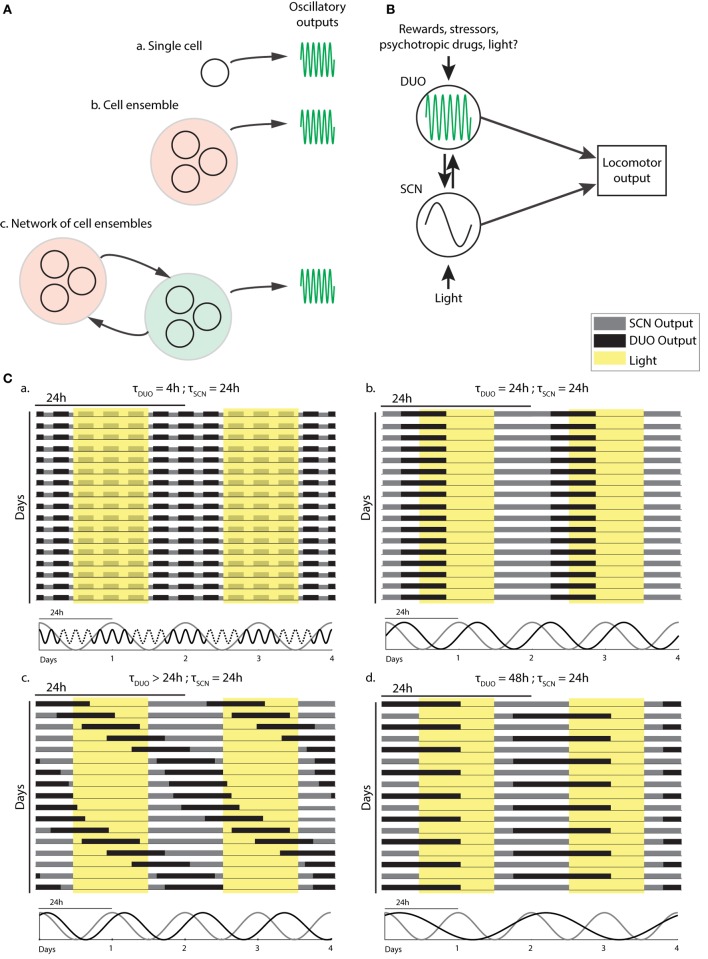
Dopaminergic ultradian oscillator (DUO) make up and output integration. **(A)** Structural basis of the DUO: ultradian rhythm generation may be cell autonomous (a), require a cell ensemble (b), or rely on a network of cell ensembles (c). **(B)** Possible DUO/circadian clock [suprachiasmatic nucleus (SCN)] interaction and output integration. LA, locomotor activity. **(C)** Schematic representation of typical LA patterns found in mice with and without dopamine system interference. The periodicities of the SCN and DUO oscillators suggested to underlie the activity patterns are illustrated below each actogram.

As DAT is only found in DA neurons and given that selective chemogenetic stimulation of DAT-expressing midbrain neurons leads to ultradian locomotor period lengthening (20), and because of the observation of striatal, extracellular dopamine fluctuating at ultradian periods, midbrain DA neurons could plausibly act as the site of ultradian locomotor rhythm generation. However, the current data are also consistent with an ultradian rhythm generator located elsewhere, which regulates extracellular dopamine levels by, for instance, rhythmic metabolic conversion, and whose period depends on dopamine tone. However, the DA degrading enzyme catechol-*O*-methyltransferase (COMT), which converts DA into 3-methoxytyramine and which is found in various brain regions including the striatum, seems not to have a significant role in clearing striatal extracellular DA upon evoked dopamine overflow based on the study of COMT deficient mice (40). As the striatum has been the site of detection of ultradian DA fluctuations (20), this finding argues against extracellular DA enzymatic conversion as a means to convey ultradian oscillator output. Interestingly, lesions to the retrochiasmatic, paraventricular, and/or arcuate nucleus regions greatly perturbs or even abolishes ultradian rhythm generation in the common voles, indicating that these brain areas either participate in rhythm generation or affect oscillator output (16, 41). Because DAT-expressing dopamine neurons are also found in the arcuate nucleus region (42, 43) and along the walls of the hypothalamic third ventricle (44), it is possible that these hypothalamic DA neurons contribute to rhythm generation as part of a network of DA neuronal populations that together make up the DUO oscillator (Figure 2A). However, selective and chronic *in vivo* activation of midbrain DA neurons using a chemogenetic strategy (20) led to a sustained lengthening of the ultradian period, suggesting that extra-midbrain DA neurons are not critical for ultradian rhythm generation/period determination.

Of note, gonadotropin-releasing hormone (GnRH) is released in a pulsatile fashion by GnRH neuron terminals at the portal vessels of the median eminence, a structure located at the base of the arcuate nucleus (45). Interestingly, serum levels of luteinizing hormone, whose release is controlled by GnRH, have been shown to fluctuate with an ultradian period of 2–3 h in male rhesus monkeys (12, 46) and luteinizing hormone levels were shown to rise about every 6 h in the mid luteal phase of the menstrual cycle in women (47). Given that the GnRH projections originating from the preoptic area traverse the retrochiasmatic area and arcuate nucleus to reach the median eminence, it is conceivable that the hypothalamic lesions affect ultradian rhythmicity in the vole by severing GnRH neuronal processes, and thus their ability to contribute to the ultradian locomotor rhythm generation by means of their role in pulse generation. However, the LH pulse frequency has been shown to differ substantially between female [1 pulse per 1 h (48)] and male [1 pulse per 2–3 h (49, 50)] mice. Because no such sexual dimorphism is reported for the ultradian locomotor periodicity, these findings argue against a key role of the GnRH pulse generator in ultradian locomotor rhythm generation. Pulsatory secretion is also a key characteristic of the hypothalamic–pituitary–adrenal axis (HPA) (51). Corticotrophin-releasing hormone (52, 53), adrenocorticotropic hormone (54, 55), as well as the glucocorticoids (CORT) (56, 57) are all rhythmically secreted into the circulation with pulse frequencies typically in the hourly range in rat (56–58) and man (59–61). Thus, as in case of GnRH/LH, also HPA axis pulse generation may not be involved in the production of ultradian locomotor rhythm which are characterized by multi-hour periodicities.

## The Methamphetamine-Sensitive (Circadian) Oscillator (MASCO) Rhythm Reflects a Specific State of the DUO

Several decades ago, it was found that treatment with the psychostimulant methamphetamine *via* the drinking water leads to the expression of a second rhythmic component in addition to the daily circadian component. Because this component exhibited periods in the circadian range (62) it was dubbed the MASCO (63). As SCN lesion (62) or genetic disruption of clock function (64) does not prevent the expression of methamphetamine-dependent rhythmicity, it was concluded that the MASCO rhythm expression does not require the known circadian clock machinery (64, 65). When methamphetamine-treated SCN-lesioned rats were given timed intraperitoneal injections with the antipsychotic haloperidol, which binds to the dopamine receptor 2 found on midbrain dopamine neurons, it shifted the rhythm phase, with the directionality of the shift depending upon the relative time point (with regard to activity onset) of haloperidol injection (66). Notably, this early finding already pointed to a critical role of dopamine in the oscillator process driving these methamphetamine-induced rhythms.

The observation that methamphetamine is not only capable of gradually lengthening the ultradian locomotor period of *Bmal1^−/−^* mice from ~4 to ≥48 h (Figure 1E) (20), but to similarly affect the ultradian oscillator in circadian intact mice, causing the 3 night-time activity peaks to transition into 2 and then 1 single peak (20) now argues that the methamphetamine-induced rhythmicity described earlier in fact represents a long period manifestation of a highly tunable ultradian oscillator, the DUO.

## Interaction of the DUO and SCN Circadian Timer

Studies on the SCN-intact common vole specifically in constant darkness showed that the ultradian rhythms in LA and feeding are phase-locked with the circadian clock, indicating coupling of the two oscillator systems (67). It was suggested that the ultradian rhythm is reset daily by the circadian clock and that it is not directly sensitive to light cues, and that phase resetting by light is instead mediated through the circadian timer. Further support for interaction between the SCN and ultradian timer comes from the observation of a phase-dependent change in oscillator speed, which is also known as relative coordination if the speed change does not lead to stable entrainment between two oscillatory processes (68, 69). For instance, under conditions of methamphetamine treatment: the second (>24 h) locomotor component frequently seems to “slow down” when overlapping with the “primary,” SCN-driven bout in methamphetamine-treated animals (63, 70) (see Figure 2C,c for illustration). In addition to an influence of the circadian clock on the ultradian oscillator, there is also evidence for the inverse: the emergence of the second long period (>24 h), likely DUO-driven component, in *DAT^−/−^* mice is associated with a simultaneous period lengthening of the SCN-driven (~24 h) component (Figure 1D, DD portion of the graph). Similar observations have been made in methamphetamine-treated animals where the SCN-component delays its phase in the presence of the second (methamphetamine dependent) component (63). Thus, it seems as if both the DUO and SCN clock produce signals for their reciprocal entrainment which may or may not lead to full entrainment between both oscillators. Of note, mice with reduced expression of DAT have been reported to exhibit a lengthened circadian LA period (71). While ultradian rhythmicity has not been explicitly probed, the authors did not rule out the possibility that the observed period lengthening could be due to the action of a dysregulated DUO as proposed by Blum et al. (20).

Genetic ablation of the orexins have been reported to attenuate the ultradian amplitude in daily locomotor behavior, heart rate, and body temperature (72), suggesting a modulating role of these peptides on DUO function. As with the monoaminergic systems, orexins and the orexin-expressing neurons are part of the ascending arousal pathway (26), receiving input from the SCN *via* the dorsomedial hypothalamic nucleus, and projecting to the midbrain area where the DA neurons reside (73). Orexins could thus serve as mediators of circadian clock control onto the DUO.

## Ultradian and Circadian Oscillator Locomotor Output Integration

The data presented in Blum et al. (20) suggest that a second oscillator is operative in the mammalian brain (Figure 2B) which fundamentally differs from the circadian timer due to its high, frequency tunability. Figure 2C illustrates how this feature can explain the profoundly deviating patterns in daily LA that are observable upon manipulation of the dopamine system.

When unchallenged, the DUO cycles at an ultradian period of, e.g., 2–4 h alongside the circadian timer, producing activity bouts throughout the 24-h cycle in voles or infants, but accounts only for the three night-time activity peaks in mice, likely due to strong daytime inhibition of DUO locomotor output by the SCN timer (Figure 2C,a). Methamphetamine treatment or DAT disruption lengthens the DUO period. This lengthening may reach 24 h, a period at which the DUO can cycle harmoniously with the SCN timer/LD cycle (Figure 2C,b). The relative phasing between the SCN timer/LD cycle and the DUO will depend on the entrainment capacity of the SCN timer/LD cycle and the free-running period of the DUO, i.e., the period the DUO would adopt in the absence of the SCN timer, e.g., the longer the DUO free-running period, the more delayed the phase of entrainment with the SCN timer/LD cycle will be (Figure 2C,b). If the SCN/LD cycle is incapable to fully entrain a long-period (>24 h) DUO, the DUO will free-run in the presence of the SCN/LD cycle; however, as a consequence of partial entrainment, its speed will be altered in a phase-dependent manner, resulting in relative coordination (Figure 2C,c). Further DUO period lengthening may lead to entrainment at 48 h likely because this frequency is again harmonious with the SCN timer/LD cycle and thus 24-h entrainment cues cause a sufficient phase shift to stably entrain the DUO at the 48-h frequency (Figure 2C,d).

## Outlook

The finding that DAT removal has a profound period lengthening effect on ultradian LA rhythms together with the discovery of synchronous fluctuations in extracellular dopamine provides a first framework for the molecular underpinnings of the oscillatory process that underlies ultradian rhythmicity. The current data indicate a central role for DA neurons in the rhythm generating process; however, it remains to be seen if rhythm generation is cell autonomous, as in case of the circadian oscillator or instead requires one or more interconnected cell ensembles (Figure 2A). Intriguingly, at least some of the LA patterns observed in rodents upon dysregulation of the dopamine system show striking similarities to the aberrant sleep:wake behavior associated with psychopathologies such as bipolar disorder (74, 75) or schizophrenia (76, 77). Given the strong concordance of LA and wakefulness for both rodents and humans (37, 78) the pattern similarities between rodent models and these human subjects indicate that the study of the DUO may have important implications in understanding the etiology of these sleep abnormalities and perhaps the psychopathologies themselves.

## Author Contributions

CB and K-FS contributed equally to the writing of this review.

## Conflict of Interest Statement

The authors declare that the research was conducted in the absence of any commercial or financial relationships that could be construed as a potential conflict of interest.
